# Development of Novel Magnetoliposomes Containing Nickel Ferrite Nanoparticles Covered with Gold for Applications in Thermotherapy

**DOI:** 10.3390/ma13040815

**Published:** 2020-02-11

**Authors:** Irina S. R. Rio, Ana Rita O. Rodrigues, Carolina P. Rodrigues, Bernardo G. Almeida, A. Pires, A. M. Pereira, J. P. Araújo, Elisabete M. S. Castanheira, Paulo J. G. Coutinho

**Affiliations:** 1 Centre of Physics (CFUM), University of Minho, Campus de Gualtar, 4710-057 Braga, Portugalecoutinho@fisica.uminho.pt (E.M.S.C.); 2 IFIMUP—Instituto de Física de Materiais Avançados, Nanotecnologia e Fotónica, Universidade do Porto, DFA-FCUP, 4169-007 Porto, Portugal

**Keywords:** magnetic/plasmonic nanoparticles, nickel ferrite, gold, magnetoliposomes, thermotherapy

## Abstract

Multifunctional nanosystems combining magnetic and plasmonic properties are a promising approach for cancer therapy, allowing magnetic guidance and a local temperature increase. This capability can provide a triggered drug release and synergistic cytotoxic effect in cancer cells. In this work, nickel ferrite/gold nanoparticles were developed, including nickel ferrite magnetic nanoparticles decorated with plasmonic gold nanoparticles and core/shell nanostructures (with a nickel ferrite core and a gold shell). These nanoparticles were covered with a surfactant/lipid bilayer, originating liposome-like structures with diameters below 160 nm. The heating capacity of these systems, upon excitation with light above 600 nm wavelength, was assessed through the emission quenching of rhodamine B located in the lipid layer. The developed nanosystems show promising results for future applications in thermotherapy.

## 1. Introduction

Nanotechnology increasingly allows the development of new techniques and strategies for therapeutic applications, such as the use of hyperthermia [1,2]. Magnetic nanoparticles are highly relevant in this regard, because of their unique properties, such as the ability to target a specific therapeutic site using external magnetic field gradients [3]. On the other hand, gold nanoparticles were employed for local heating of cells, focusing on thermotherapy [4,5,6,7,8]. The localized surface plasmon resonance (LSPR) exhibited by metal nanoparticles depends on the size, shape, and dielectric constant of the surrounding environment [8,9]. Owing to the large absorption cross-sections, plasmonic nanoparticles can produce significant heating and, hence, can be used to increase local temperatures [8,9,10]. Therefore, plasmonic nanoparticles, mainly gold-based ones owing to their biocompatibility, found a wide range of applications in different technological areas, such as biosensors, clinical methods, immunology assays, photothermolysis of tumor cells, detection and control of microbial species, as vehicles for drugs, and in monitorization of cells [4,5,6,7,8,9,10].

Considering magnetic nanoparticles, particles with superparamagnetic properties are preferred in biomedicine, as they exhibit a notable magnetization only by the application of an external magnetic field, with no remnant magnetization upon magnetic field removal [11,12,13,14]. Generally, nickel ferrite nanoparticles are superparamagnetic when their size is smaller than a critical diameter of around 30 nm [15]. However, nickel-based nanoparticles present some issues such as potential toxicity, high reactivity, and easy degradation, due to the high surface/volume ratio [16]. In order to overcome these problems and make them suitable for biological applications, nickel-containing nanoparticles are usually protected by a suitable coating, such as lipids, polymers, or silica [15,17,18,19].

Combined therapies provide a promising solution for addressing tumor heterogeneity and drug resistance issues, exploring synergistic effects of the different mechanisms of action of multiple therapies, and achieving multiple targets. Hereby, magnetic/plasmonic nanoparticles afford a high therapeutic potentiality owing to the combination of different strategies, such as photothermia, photodynamic therapy, magnetic hyperthermia, and magnetic-guided drug delivery [19]. In this context, iron-oxide magnetic nanoparticles covered with gold or carbon were used [20,21,22]. Recently, manganese ferrite/gold core/shell nanoparticles entrapped in lipid bilayers were also developed as nanocarriers for antitumor drugs [23].

Considering these advantages, in this work, both core/shell nickel ferrite/gold nanoparticles and gold-decorated nickel ferrite nanoparticles were prepared. These nanoparticles were covered with a surfactant/lipid bilayer, forming the so-called solid magnetoliposomes. The local heating capacity of these nanosystems was assessed through the inhibition of fluorescence of a fluorophore linked to the lipid layer, when the systems were excited with a light source. The new nanosystems exhibit a strong potential to promote local heating, making them promising for photothermia applications.

## 2. Materials and Methods

In all preparations, spectroscopic-grade solvents and ultrapure water of Milli-Q grade (MilliporeSigma, St. Louis, MO, USA) were used.

### 2.1. Preparation of Nickel Ferrite/Gold Nanoparticles

#### 2.1.1. Preparation of Nickel Ferrite Nanoparticles

Nickel ferrite nanoparticles were prepared by a co-precipitation route in a 5 mL aqueous solution, by reacting 1 mL of nickel chloride aqueous solution (1 M) and 2 mL of FeCl_3_ ·6H_2_O solution (1 M) with 1.818 mL of sodium hydroxide solution (18.94 M). After 40 min at 80 °C, under magnetic stirring, nickel ferrite nanoparticles were formed [15]. The nanoparticles (NPs) were washed to eliminate possible reactants in excess. After drying, the obtained NPs were calcined as previously described [15]. After calcination, in order to remove non-magnetic particles and possible impurities, a new washing process was performed, through successive centrifugation and magnetic decantation.

#### 2.1.2. Preparation of Gold Nanoparticles

A stable solution of gold nanoparticles in toluene was prepared following a method similar to that described by Brust et al. [24], which uses a series of tetraalkylammonium bromides, R_4_N^+^Br^−^ (with R representing alkyl chains ranging from C_6_ to C_18_ length), as phase-transfer reagents. Then, 1.5 mL of an aqueous solution of gold(III) chloride hydrate, HAuCl_4_ (30 mM), was mixed with tetraoctylammonium bromide (TOAB) in toluene (4 mL of 50 mM). The two-phase mixture was vigorously stirred, until all the tetrachloroaurate was transferred into the organic phase, which turned deep orange in color, while the aqueous phase became colorless. Maintaining stirring, aqueous sodium borohydride (1.5 mL of freshly prepared 0.4 M solution) was slowly added. Within a few seconds, the orange color of the organic phase changed to ruby red. After further stirring for 20 min, the organic phase was extracted and washed five times with deionized water.

#### 2.1.3. Preparation of Nickel Ferrite Nanoparticles Decorated with Gold

The synthesis of nickel ferrite nanoparticles decorated with gold nanoparticles was performed using a coupling agent, 1,1′-carbonyldiimidazole (CDI). Thus, 1 mg of magnetic nanoparticles were dispersed in 5 mL of previously dried 1,4-dioxane. To this suspension, CDI was added, corresponding to a five-fold excess over the ferrite number of moles. The suspension was heated at 60 °C and kept warmed and closed in a sonication bath. The nanoparticles were then washed with absolute ethanol using successive centrifugations at 3500 rpm for 5 min, to remove unbound CDI. Then, cysteamine was added corresponding also to a five-fold excess over the number of moles of ferrite. The tubes were kept at 60 °C for 1 h. Finally, the nanoparticles were washed with ethanol by successive centrifugations at 3500 rpm for 5 min, and dried in an oven. The cysteamine- functionalized nickel ferrite nanoparticles were dried in an oven, and 1 mg was redispersed in toluene. Then, 160 µL of gold nanoparticles (NPs) diluted in 3 mL of toluene were added, resulting in a color change. When using a small magnet, the color disappeared as the magnetic particles accumulated on the wall of the tube. This behavior indicates the successful coupling of gold NPs to the SH groups of cysteamine molecules covalently linked to the surface of nickel ferrite nanoparticles. These particles were magnetically decanted and washed with ethanol to remove unbound gold nanoparticles and released TOAB molecules. The supernatant was always without visible color; thus, it is reasonable to assume that all gold NPs were bound to nickel ferrite NPs.

#### 2.1.4. Preparation of Nickel Ferrite/Gold Core/Shell Nanoparticles

Core/shell nanoparticles, with a nickel ferrite core and a gold shell, were prepared from the growth of a gold shell around the gold-decorated nickel ferrite nanoparticles. For that, a procedure adapted from a previously reported method (“seeding”) was used [25]. Specifically, gold-decorated NiFe_2_O_4_ (acting as gold seeds) dispersed in 1.5 mL of water (gold concentration of 0.6 mM) and magnetically decanted was redispersed in an aqueous mixture of 2.4 mL of 0.01% HAuCl_4_ and 100 μL of 40 mM hydroxylamine. Absorption spectra were taken in 10 s intervals. Then, a further amount of gold ions was added (4.2 times more than before), by using 100 μL of 1% HAuCl_4_ solution, and absorption spectra were acquired at 10 s intervals. At the end, the particles were magnetically decanted, washed with water, and redispersed in ethanol.

The stability of nanoparticles dispersions in phosphate-buffered saline (PBS) medium (pH = 7.0) (with the same NP concentration used in the preparation of magnetoliposomes) was evaluated by following the UV/Visible absorption for 1 h.

### 2.2. Preparation of Solid Magnetoliposomes

Solid magnetoliposomes (SMLs) labeled with a fluorophore were prepared using the phospholipid DOPG (1,2-dioleoyl-*sn*-glycero-3-phospho-rac-(1-glycerol) sodium salt) (one of the components of the plasma membrane and of the pulmonary surfactant) and the labeled lipid Rhodamine B-DOPE (1,2-dioleoyl-*sn*-glycero-3-phospho-ethanolamine-*N*-lissamine rhodamine B sulfonyl (ammonium salt)), from Avanti Polar Lipids, Alabaster, AL, USA.

A solution of functionalized nickel ferrite/gold nanoparticles (with cysteamine as a bridge) in absolute ethanol (1.5 mL) was prepared. Then, octadecylamine (ODA) in a five-fold excess was added to the nanoparticles, and this solution was stirred for 1 h. The solution was washed with successive centrifugations and magnetic decantation to remove unbound ODA. After this step, an ethanolic solution of 4:1 DOPG and Rhodamine-DOPE, at 5 × 10^−5^ M total lipid concentration, was added to the nanoparticles covered with ODA. After mixing followed by evaporation of the solvent, a uniform lipid film was obtained, ultrapure water was added, and sonification was used to promote lipid rearrangement and magnetoliposome formation, followed by two washing steps again consisting of centrifugation and magnetic decantation.

### 2.3. Spectroscopic Measurements

Absorption spectra were carried out in a Shimadzu UV-3600 Plus UV–Vis–near-infrared (NIR) (Shimadzu Corporation, Kyoto, Japan) spectrophotometer. For fluorescence emission assays, a Horiba Fluorolog 3 spectrofluorimeter (HORIBA Jobin Yvon IBH Ltd., Glasgow, UK) was used, with double monochromators in both excitation and emission, Glan–Thompson polarizers, and a temperature-controlled cuvette holder. All spectra were corrected for the instrumental response.

Förster resonance energy transfer (FRET) assays were performed to confirm the formation of the surfactant/lipid bilayer in SMLs. In these assays, the dye Nile Blue was incorporated in the first (surfactant) layer, while the fluorescent lipid Rhodamine B-DOPE (1,2-dioleoyl-*sn*-glycero- 3-phospho-ethanolamine-*N*-lissamine Rhodamine B sulfonyl (ammonium salt)) (from Avanti Polar Lipids, Alabaster, AL, USA) was included in the second (lipid) layer. FRET efficiency, corresponding to the proportion of donor (Rhodamine B) molecules that transferred their energy to the acceptor (Nile Blue) molecules, was determined through donor emission quenching, as previously described [23,26]. The distance between donor and acceptor molecules was estimated through FRET efficiency, after calculation of the Förster radius (*R*_0_) and fluorescence quantum yield of the donor $\left(\Phi_{D}^{0}\right)$ [26].

### 2.4. Structural and Magnetic Characterization

High-resolution transmission electron microscopy (HR-TEM) images were obtained in a JEOL JEM 2010F microscope operating at 200 kV, coupled to an electron-dispersive spectroscopic (EDS) analyzer (JEOL Ltd., Tokyo, Japan) at C.A.C.T.I (Centro de Apoio Científico e Tecnolóxico á Investigación), Vigo, Spain. A drop of the sample was placed onto a copper grid with Formvar/carbon (Agar Scientific Ltd., Essex, UK) and left to dry. TEM images were processed by ImageJ software (National Institutes of Health (NIH), version 1.52p, Bethesda, MD, USA), by enhancing local contrast followed by an automatic local thresholding and particle analysis. Particle diameters were estimated using the area of each particle, and the corresponding histogram was fitted to a Gaussian distribution.

The magnetoliposome (lipid concentration: 1 mM) mean diameter and size distribution were determined by dynamic light scattering (DLS) in a NANO ZS Malvern Zetasizer equipment (Malvern Panalytical Ltd., Malvern, UK) at 25 °C, using an He-Ne laser of λ = 632.8 nm and a detector angle of 173°. Five independent measurements were performed for each sample.

X-ray diffraction (XRD) measurements were obtained in a conventional PAN’alytical X’Pert PRO diffractometer (Malvern Panalytical Ltd., Malvern, UK) diffractometer, operating with CuK_α_ radiation in a Bragg–Brentano configuration.

Magnetic measurements were carried out at room temperature in a superconducting quantum interference device (SQUID) magnetometer Quantum Design MPMS5XL (Quantum Design Inc., San Diego, CA, USA), using applied magnetic fields up to 5.5 T.

### 2.5. Assessment of the Photothermal Effect

**Figure 1:**
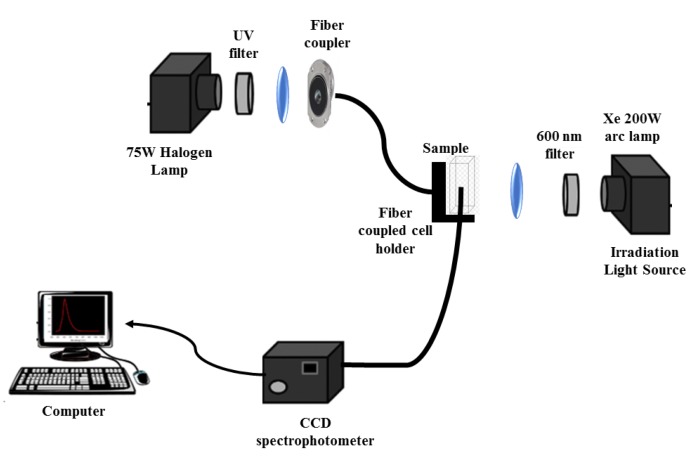
Experimental set-up for measurements of the photothermal effect.

An irradiation set-up, prepared for this purpose (Figure 1), comprised a xenon arc lamp (200 W) and an optical fiber, using a Thorlabs FEL0600 (Thorlabs Inc., Newton, NJ, USA) long-pass filter with cut-on wavelength at 600 nm, to guarantee the excitation of only the gold nanoparticles (not exciting Rhodamine B dye). Magnetoliposomes including the labeled lipid Rhodamine B-DOPE were irradiated, and the fluorescence of Rhodamine B was monitored as a function of time. The monitoring channel consisted of a 75 W halogen bulb source coupled to an optical fiber, and the emission was collected at 90° geometry, using a compact charge-coupled device (CCD) optical fiber spectrophotometer (350–700 nm, Δλ < 0.5 nm, slit = 20 µm, CCS100 Thorlabs Inc., Newton, NJ, USA), with a 60 s acquisition time under dark conditions. In order to avoid the impact of scattered light from the irradiation lamp, the irradiation optical channel was blocked during the acquisition of the emission spectra.

## 3. Results and Discussion

Considering the main objective of this work, i.e., the development of magnetoliposomes based on improved magnetic/plasmonic nanoparticles for applications in phototherapy, optimization of the synthesis procedures of the several components of the whole nanosystem was pursued.

### 3.1. Nanoparticles Synthesis and Characterization

#### 3.1.1. Synthesis of Magnetic/Plasmonic Nanoparticles

**Figure 2:**
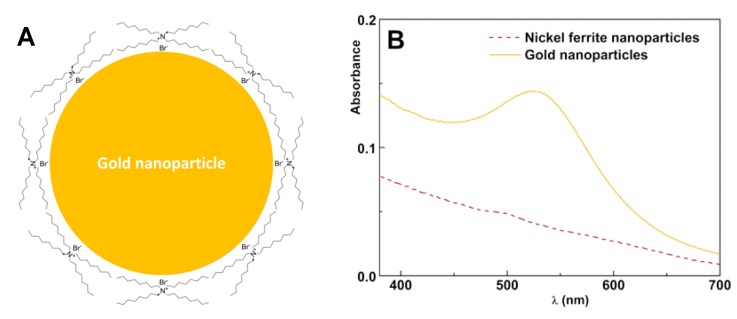
(**A**) Schematic representation of the interactions between R_4_N^+^ and Br^−^ species adsorbed on the gold nanoparticle surface. (**B**) Absorption spectra of nickel ferrite nanoparticle and gold nanoparticle dispersions.

The nickel ferrite nanoparticles were prepared as previously reported [15], being subjected to a calcination step at 800 °C for 2 h, to improve crystallinity. The gold nanoparticles were obtained by the Brust–Schiffrin method [24,27], using TOAB as a phase-transfer agent to the organic phase. TOAB promotes colloidal stabilization of the gold nanoparticles by anchoring of alkyl chains due to the attachment of R_4_N^+^Br^−^ ion pairs to the gold nanoparticle surface, as proposed by Fink et al. [27] (Figure 2A). Absorption spectra of the synthesized nickel ferrite and gold nanoparticles are shown in Figure 2B.

The prominent plasmon resonance band, centered at 522 nm, confirmed the formation of spherical gold nanoparticles. In aqueous medium (with refractive index *n* = 1.333), the position of the plasmon band would correspond to a size near 20 nm [25]. As toluene has a higher refractive index (*n* = 1.497), the plasmon band position is shifted to lower energy, such that the prepared nanoparticles are expected to be much smaller. Lee et al. [28] showed that the sensitivity of the gold plasmon band position to the medium was 331, 238, and 154 nm/RIU (RIU = refractive index units) for, respectively, 20-, 40-, and 60-nm-sized gold nanospheres. For 15-nm gold NPs, capped with a positive surfactant (cetyltrimethylammonium bromide, CTAB), an experimental value of 44 nm/RIU was reported [29] using water/glycerol mixtures. Reported HR-TEM results on gold particles obtained by the same procedure [24] were between 3 nm and 5 nm. For the latter in water, a value of 515 nm was published [30]. Thus, taking a sensitivity of 40 nm/RIU, a value of 521.5 nm can be predicted for 5-nm gold NPs in toluene.

**Figure 3:**
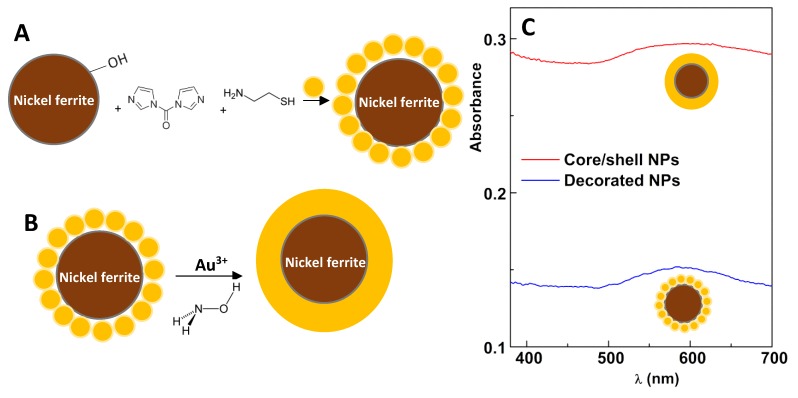
(**A**) Schematic representation of the synthesis of gold-decorated nickel ferrite nanoparticles dispersed in ethanol. (**B**) Schematic representation of the synthesis of core/shell nickel ferrite/gold nanoparticles by “seeding” method. (**C**) Absorption spectra of nickel ferrite nanoparticles decorated with gold nanoparticles and nickel ferrite/gold core/shell nanoparticles.

The coupling of magnetic nanoparticles to gold nanoparticles, to obtain nickel ferrite nanoparticles decorated with gold nanoparticles, was carried out using the coupling reagents 1,1′-carbonyldiimidazole (CDI) and cysteamine. CDI promotes the activation of –OH surface groups of nickel ferrite, which bind the amine moieties of cysteamine molecules (NH_2_CH_2_CH_2_SH), providing the surface of magnetic NPs with thiol groups that exhibit a strong affinity to gold, producing gold-decorated magnetic nanoparticles (Figure 3A). These gold-decorated nanoparticles serve as support for the growth of a shell of gold, through the use of Au^3+^ ions and hydroxylamine (Figure 3B). This method (“seeding method”) consists of the reduction of Au^3+^ cations by hydroxylamine, with the reaction being accelerated in the presence of gold surfaces [25] (Figure 3B). No new nanoparticle nucleation occurs in solution, and all the added gold is used to enlarge already existing nanoparticles [25]. 

Absorption spectra (Figure 3C) evidenced the gold resonance plasmonic band of gold-decorated nickel ferrite nanoparticles, centered at 580 nm. The red-shifted and enlarged LSPR band resulted from the near proximity of gold NPs [31] when attached to the nickel ferrite surface. Assuming that all 5-nm gold NPs were attached to the nickel ferrite surface (0.177 mg of gold), an estimation of 1.2 gold NPs per particle could be made (using ρ_Au_ = 19.4 g/cm^3^ and ρ_NiFe2O4_ = 5.4 g/cm^3^). This value was not compatible with the significant red shift of the plasmon band that was observed. If cysteamine-functionalized nickel ferrite nanoparticles form clusters in toluene, then, assuming one layer compact packaging (12 neighbours, 0.74 packaging density), a value of 23.9 gold NPs per particle, from a maximum of 226, could be predicted (35.5 nm cluster size). This estimation would change to 110.8 in 566 when two covering compact layers are considered (57.5 nm cluster size). These values are consistent with close proximity effects on optical properties of gold NPs. Additional support for the aggregation of cysteamine-functionalized nickel ferrite nanoparticles is the fact that –SH groups easily oxidize in the presence of oxygen, forming S–S bridges.

The growth of the gold shell further broadened the plasmon band, making its maximum undefinable. This observation is in accordance with a similar system, recently reported by our group [32], in which a gold shell was grown on manganese ferrite NPs, but using a distinct synthesis strategy. It seems then that the coupling and seeding procedures generated NiFe_2_O_4_ clusters covered by a hopefully continuous gold layer.

**Figure 4:**
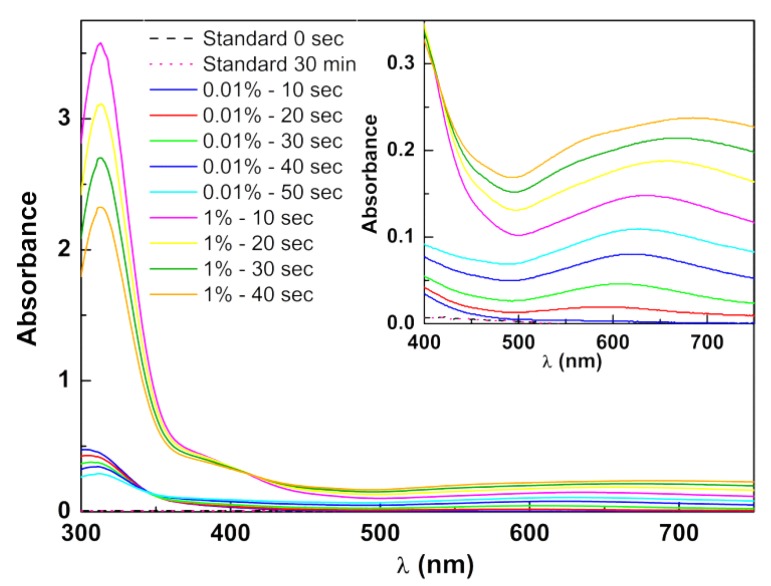
Absorption spectra of nickel ferrite/gold core/shell nanoparticles with increasing time, after addition of hydroxylamine and chloroauric acid (0.01% and 1%).

Figure 4 evidences the rise in the SPR band of gold with the addition of Au^3+^, giving support for the growth of a gold shell surrounding nickel ferrite nanoparticles or, most probably, nickel ferrite clusters. With 0.01% chloroauric acid (HAuCl_4_), the effect was low, differently from what was reported by Brown et al. [25]. In their work, 50 µL of a 17 nM solution of 12-nm spherical gold seeds ([Au^0^]_initial_ = 0.017 mM) was used, whereas 1.5 mL of gold-decorated nickel ferrite was used here, containing [Au] = 0.6 mM ([Au^0^]_initial_ = 0.36 mM). In order to increase the deposition of gold and try to obtain a continuous gold shell, 1% HAuCl_4_ was used, resulting in a further increase in the characteristic plasmon band of gold (Figure 4).

**Figure 5:**
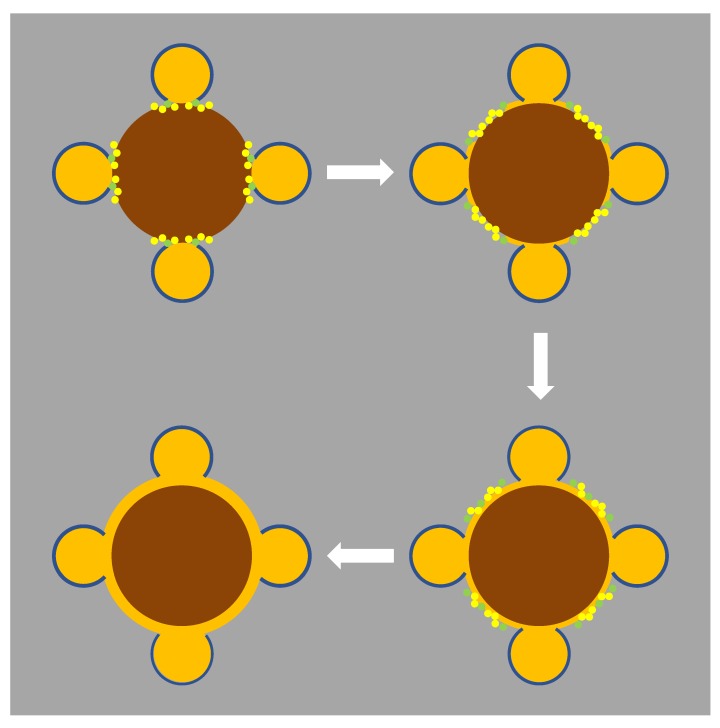
Proposed mechanism of gold growth by seeding method in gold-decorated nickel ferrite nanoparticles. The brown circles represent NiFe_2_O_4_ clusters; orange circles represent gold nanoparticles; the blue layer represents the tetraoctylammonium bromide (TOAB) molecules layer; small yellow circles are AuCl_4_^−^ molecules; small green circles are hydroxylamine molecules.

It is interesting to note that, following the second addition of HAuCl_4_, a change in the isosbestic point occurred. At those points, the molar absorptivity of HAuCl_4_ was the same as that of gold NPs. This variation is an indication of a change in the type of growth of the gold layer. As gold NPs were covered by positive TOAB molecules, which have very low solubility in water, the accessibility of gold ions was limited and would mainly occur near the region of close contact with the surface of NiFe_2_O_4_. The reason for this is that, upon nanoparticle coupling, TOAB molecules were displaced by SH groups from cysteamine that was covalently bound to the NiFe_2_O_4_ nanoparticles. Thus, the gold growth would occur mainly near the contact with nickel ferrite spreading over its surface. The second stage could then correspond to growth of the resulting thin continuous layer. The proposed mechanism is depicted in Figure 5.

The amount of deposited gold can be estimated by the decrease in the absorption band near 320 nm, although some rising contribution from deposited gold also appeared at this wavelength. Considering the complete deposition of the initial HAuCl_4_ and the observed 35% decrease in the second state, a 0.34-mg total amount of deposited gold could be estimated (upon completion, the value would be 0.73 mg). The quantities needed for a 1-nm gold shell over nickel ferrite, as individualized NPs or as 1|2 layer compact clusters, would be 1.1 mg or 0.44 mg|0.26 mg, respectively. For a 2-nm shell, the amount would change to 2.6 mg or 0.95 mg|0.54 mg. Overall, the results seem to be compatible with a 1–2-nm gold shell over NiFe_2_O_4_ clusters.

The stability of both gold-decorated and core/shell NPs dispersions in PBS buffer (pH = 7.0) was also evaluated. From UV–Vis absorption measurements over time (Figure S1, Supplementary Materials), it can be observed that the nanoparticles dispersions were stable for 1 h, with no evident sedimentation. 

#### 3.1.2. XRD Analysis

**Figure 6:**
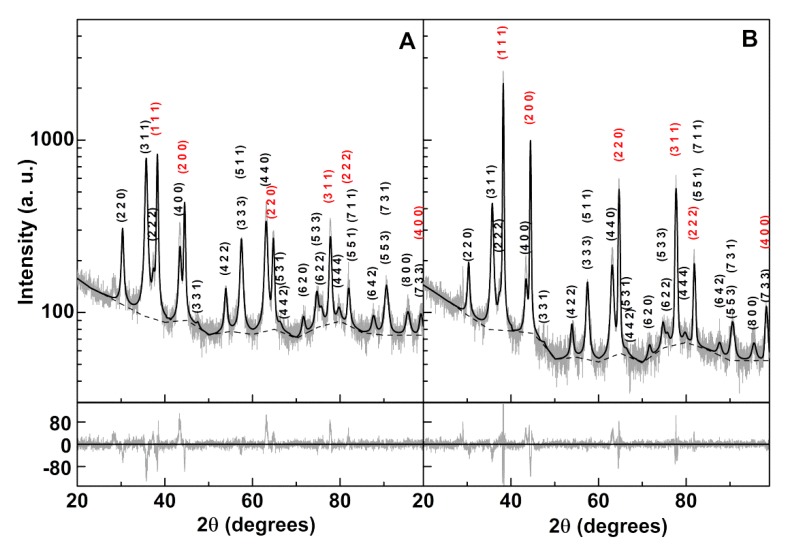
X-ray diffraction pattern of (**A**) nickel ferrite nanoparticles decorated with gold nanoparticles, and (**B**) core/shell nickel ferrite/gold nanoparticles. Gray lines: experimental patterns; black lines: fitted patterns. Background: short-dashed lines. Miller indices: black—nickel ferrite; red—gold.

X-ray diffraction (XRD) results for the prepared gold conjugated nickel ferrite nanoparticles are shown in Figure 6. The presence of well-defined peaks indicates a highly crystalline structure. Diffraction peaks of gold were observed at 2θ = 38.2° (1 1 1), 44.4° (2 0 0), 64.6° (2 2 0), 77.6° (3 1 1), 81.8° (2 2 2), and 98.2° (4 0 0), corresponding to CIF 9013035 (space group Fm-3m), both for the gold-decorated (Figure 6A) and gold shell (Figure 6B) structures. Interestingly, these peaks more than doubled in intensity upon the seeding process that was expected to originate gold shell formation. That huge increase indicates a substantial enhancement of the gold content, thus supporting the growth of the existing gold-decorated particles, hopefully filling the gaps and leading to a continuous gold shell. Diffraction peaks of the NiFe_2_O_4_ crystalline structure (Figure 6) were observed at 2θ = 30.3° (2 2 0), 35.7° (3 1 1), 37.4° (2 2 2), 43.4° (4 0 0), 47.5° (3 3 1), 53.8° (4 2 2), 57.4° (3 3 3) and (5 1 1), 63.1° (4 4 0), 66.3° (5 3 1), 67.4° (4 4 2), 71.6° (6 2 0), 74.6° (5 3 3), 75.7° (6 2 2), 79.7° (4 4 4), 85.6° (6 4 2), 82.6° (5 5 1) and (7 1 1), 87.6° (6 4 2), 90.5° (7 3 1) and (5 5 3), 95.4° (8 0 0), and 98.4° (7 3 3), adapted from CIF file 2300618 (MnFe_2_O_4_, space group Fd-3m, replacing Mn with Ni and considering a totally inverted spinel structure [33]).

Rietveld analysis was performed using FullProf software suite (version 5.8, J. Rodríguez-Carvajal, Lab. Léon Brillouin, Gif sur Yvette, France) [34], with a background defined by linear interpolation between a set of points at constant scattering angles and refinable intensities. The calculated parameters and phase sizes are presented in Table 1.

The nickel ferrite phases had an average size of approximately 11.5 nm, which is similar to what was obtained in a previous work [15]. The gold phase in the decorated NiFe_2_O_4_ particles (Au@NiFe_2_O_4_) was estimated as 22.7 nm. This is in contrast with what was reported through HR-TEM results on gold particles obtained using the same procedure [24] and from size estimation, made from the plasmon absorption band (in the previous section), which was 5 nm. Even considering that the gold phase size corresponded to the double of its diameter (a given X-ray when interacting with a decorated nanoparticle encounters, on average, two gold NPs) [4,32], the estimated size was still, at least, two times larger than expected. This means that specific structural effects occurred in the diffraction of X-rays by gold nanoparticles distributed over the surface of NiFe_2_O_4_. This is in line with other studies for gold shells covering magnetite nanoparticles where peculiar broadening effects were observed for the gold phase; specifically, the diffraction peak width for the 10-nm magnetite core was similar to that for the 2-nm gold shell [35]. Those structural effects were expected to also influence the diffraction peak intensity; thus, no reliable information could be taken from the mass percentage estimated by FullProf. For the particles that originated from the gold decorated NiFe_2_O_4_ upon gold phase growth (NiFe_2_O_4_/Au), the already mentioned increase in gold phase diffraction intensity corresponded to an increase in gold content of 2.74 times. Albeit an overestimation, as nickel ferrite diffraction peaks were seen to slightly decrease in intensity, this was compatible with the calculated deposited gold amount estimated in previous section, considering nickel ferrite clusters of 1|2 layers: a 1 nm gold shell results from an increase from 0.18 mg to 0.44 + 0.18|0.26 + 0.18 mg ⇔ 3.4|2.4; a 2 nm gold shell results from an increase from 0.18 mg to 0.95 + 0.18|0.54 + 0.18 mg ⇔ 6.2|4.0. The size estimation of the gold phase was now 59.6 nm. Again, this value is an overestimation, as several studies reported that gold shell thicknesses above 2 nm completely eliminated core diffraction peaks [35,36]. In another work, a huge diffraction intensity decrease from the core was observed with a gold shell up to 8.5 nm [37]. As the intensity of NiFe_2_O_4_ core diffraction peaks slightly decreased, the XRD results were compatible with a shell of ~1 nm covering the nickel ferrite surface (Figure 6).

#### 3.1.3. Transmission Electron Microscopy (TEM)

**Figure 7:**
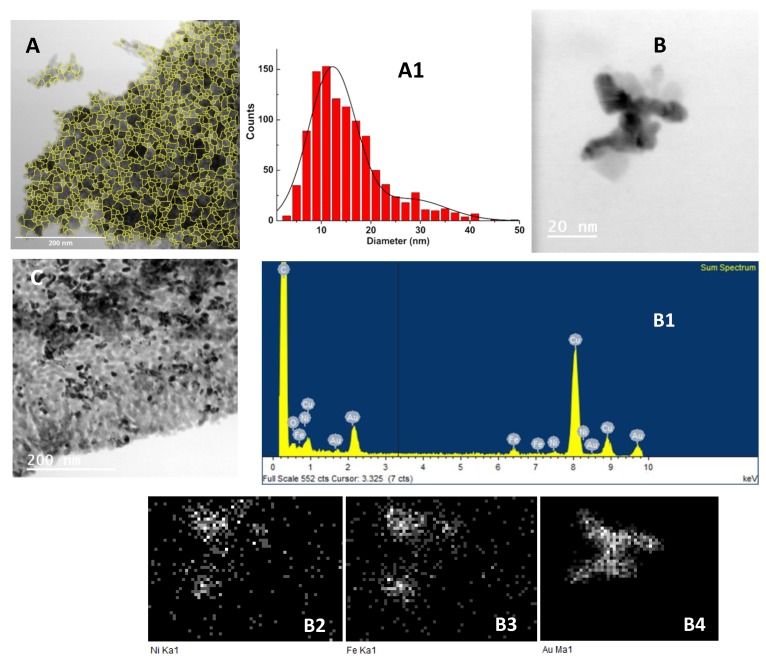
TEM images of the nickel ferrite/Au nanoparticles. (**A**) Nickel ferrite nanoparticles decorated with gold nanoparticles (scale: 200 nm). (**A1**) Size histogram of image A and fitting to a Gaussian distribution. (**B**) Nickel ferrite nanoparticles decorated with gold nanoparticles (scale: 20 nm). (**B1**) Electron-dispersive X-ray (EDX) spectrum of TEM image B, using Ni (**B2**), Fe (**B3**), and Au (**B4**) X-ray emission lines. (**C**) Core/shell nickel ferrite/gold nanoparticles (scale: 200 nm).

TEM images of the gold decorated nickel ferrite nanoparticles are presented in Figure 7A,B. Small dark spots above diffuse particles (Figure 7A) are an indication of gold nanoparticles on small clusters of nickel ferrite. Using ImageJ software (version 1.52p, National Institutes of Health (NIH), Bethesda, MD, USA), it was possible to isolate the nanoparticles in Figure 7A by inversion, background subtraction, and morphological segmentation through MorphoLibJ plugin [38] (morphological type gradient = 5; tolerance = 10; connectivity = 4), taking the “watershed lines” as particle outlines. The result is shown in Figure 7A, and the corresponding perimeter-derived size histogram is represented in Figure 7A1. Using a Gaussian distribution fit, two populations were obtained, with sizes of 12 ± 5 nm and 28 ± 7 nm, which corresponded, respectively, to nickel ferrite nanoparticles that the segmentation procedure was able to isolate and to nickel ferrite aggregates. 

Figure 7B evidences an aggregated structure with dark zones, and the corresponding electron-dispersive X-ray (EDX) spectra and elemental maps (Figure 7B1–B4) demonstrate the presence of gold in those dark areas, where the diffuse regions correspond to nickel ferrite. Upon the “seeding” procedure (Figure 7C), it was observed that the dark zones increased, indicating a higher coverage of the nickel ferrite NPs. The observed fact that some of the particles did not have dark zones indicated that efficiency of the used experimental procedures was far from 100%. This would predict higher gold shell thicknesses than the estimations in the previous sections.

#### 3.1.4. Magnetic Measurements

Nickel ferrite is supposed to adopt an inverse spinel structure, with Ni^2+^ ions on octahedral B sites, denoted as O_h_-sites, and Fe^3+^ ions equally distributed on both the tetrahedral A sites (T_d_-sites) and O_h_-sites, which is supported by estimations of energies of formation that favor the reverse spinel rather than spinel structure [39]. However, NiFe_2_O_4_ nanoparticles were found to exhibit a mixed spinel structure together with the reverse spinel [40,41], as some Ni^2+^ cations may occupy the T_d_ sites.

A general formula for a nickel ferrite structure is (Ni_1*− x*_Fe*_x_*)[Ni*_x_*Fe_2 − *x*_]O_4_, where *x* is the degree of inversion. The magnetic moments arise from the local moments of the Ni^2+^ with 3*d*^8^ electrons and Fe^3+^ with 3*d*^5^ electrons. The net magnetization comes from the Ni^2+^ (O_h_) cations alone, ~2μ_B_, whereas the Fe^3+^ moments, ~5μ_B_, in a high spin state for both the O_h_ and T_d_ sites, are antiparallel and cancel each other. This leads to an overall magnetic moment of 2μ_B_ per formula unit (FU) [42]. Density functional theory (DFT) calculations yielded a consistent result for the magnetic moment of nickel ferrite with inverse spinel structure, ~2μ_B_/FU, corresponding to a saturation magnetization, *M*_s_, of 50 emu∙g^−1^ [39].

**Figure 8:**
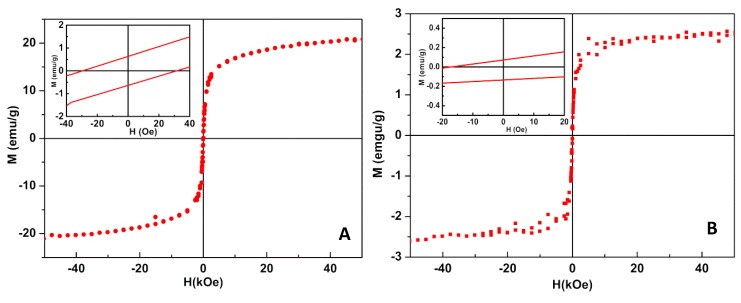
Magnetization hysteresis loops of magnetic/plasmonic nanoparticles at room temperature. (**A**) NiFe_2_O_4_ nanoparticles decorated with Au nanoparticles. (**B**) NiFe_2_O_4_/Au core/shell nanoparticles. Insets: Enlargement of the loop in the low field region.

The magnetic properties of NiFe_2_O_4_/Au NPs were assessed by the magnetic hysteresis loop (Figure 8), which evidences the relationship between the induced magnetic moment and the applied magnetic field. Both types of magnetic/plasmonic NPs displayed a superparamagnetic behavior, as the ratio between remnant magnetization, *M*_r_, and saturation magnetization, *M*_s_ (Table 2), was lower than 0.1 [42,43]. 

The saturation magnetization of the gold-decorated nanoparticles was only slightly lower than that previously reported for nickel ferrite nanoparticles obtained via the coprecipitation method [15], showing that gold nanoparticles at the nickel ferrite surface were indeed small and/or an incomplete coverage of magnetic nanoparticles occurred. The low saturation magnetization value for NiFe_2_O_4_/Au core/shell NPs was due to the presence of a diamagnetic gold layer. 

### 3.2. Magnetoliposome Synthesis and Characterization

#### Synthesis

An innovative synthesis of solid “magnetoliposomes” was developed. Firstly, ODA was used to cover the nickel ferrite/gold nanoparticles, increasing interactions with the surface of gold through the amine group of ODA molecule. The nanoparticles coupled to ODA were then subsequently covered with a DOPG lipid layer. 

**Figure 9:**
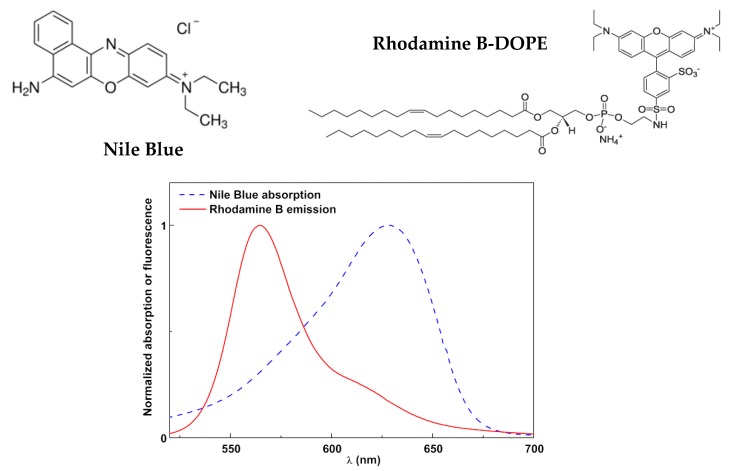
Above: Structure of the dye Nile Blue and the labeled lipid Rhodamine B-DOPE (1,2-dioleoyl-*sn*-glycero-3-phospho-ethanolamine-*N*-lissamine rhodamine B sulfonyl (ammonium salt)). Below: Spectral overlap between Rhodamine B emission and Nile Blue absorption, confirming the possibility of Förster resonance energy transfer (FRET).

To prove the surfactant/lipid bilayer structure around the magnetic/plasmonic nanoparticles, the FRET process was used. For that, the dye Nile Blue (Figure 9) was used to label the ODA inner layer, taking advantage of its amine group for gold binding. This dye forms a suitable donor–acceptor pair with Rhodamine B in FRET assays, with the latter being the energy donor and Nile Blue being the energy acceptor (Figure 9). Rhodamine B was included in the DOPG lipid layer through the labeled lipid Rhodamine B-DOPE (Figure 9).

**Figure 10:**
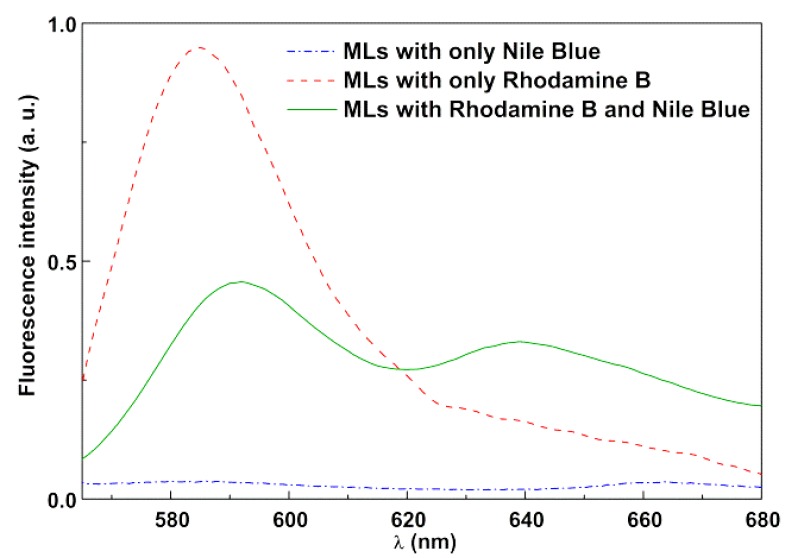
Fluorescence spectra (λ_exc_ = 510 nm) of magnetoliposomes (MLs) labeled with only Rhodamine B-DOPE, labeled with only Nile Blue, and labeled with both Rhodamine B-DOPE and Nile Blue.

The fluorescence emission spectra of magnetoliposomes containing only the Nile Blue dye (acceptor), containing only Rhodamine B-DOPE (Rhodamine as energy donor), and containing both dyes are shown in Figure 10, exciting only the donor (λ_exc_ = 510 nm). The spectrum with only the donor exhibited a prominent Rhodamine B emission band, while, for magnetoliposomes containing only Nile Blue, the emission was negligible.

The fluorescence spectrum of the system containing both donor and acceptor evidenced a notable decrease in the Rhodamine B emission and the prominent appearance of Nile Blue fluorescence, confirming the occurrence of non-radiative energy transfer from the excited Rhodamine B moiety (in the labeled lipid) to the dye Nile Blue. A FRET efficiency of 52% was estimated, corresponding to a donor–acceptor distance of 4.60 nm, with *R_0_* being calculated as 4.65 nm and $\Phi_{D}^{0}$ = 0.13 (value for Rhodamine B-DOPE in liposomes determined by the standard method [44,45], using as reference Rhodamine B in basic ethanol with Φ_F_ = 0.65 at 25 °C [46]). This distance proved the formation of a liposome-like structure, considering the reported cell membrane thickness in the range 7–9 nm [47]. 

**Figure 11:**
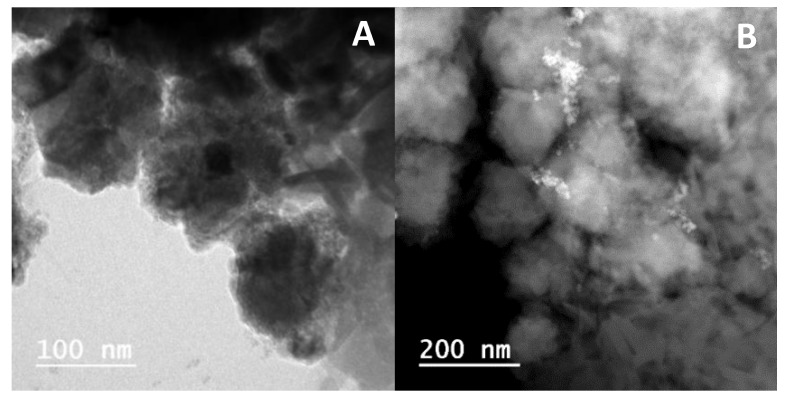
TEM images of magnetoliposomes containing magnetic/plasmonic NiFe_2_O_4_/Au nanoparticles: (**A**) Decorated nanoparticles. (**B**) Core/shell nanoparticles.

TEM images of these nanosystems containing both types of nanoparticles are shown in Figure 11. Generally, spherical structures, with sizes around or slightly above 100 nm were observed in the two cases. To guarantee an enhanced permeability and retention (EPR) effect of loaded drugs, the size of (magneto)liposomes needs to be small. Moreover, successful extravasation into tumors was reported to occur for nanosystems with diameters lower than 200 nm [48], which was the case here, as revealed by the TEM images. Therefore, regarding size, the developed nanosystems are suitable for therapeutic applications.

The values of hydrodynamic diameter determined by DLS are in accordance with TEM data for both types of NPs (Table 3). Magnetoliposomes containing nickel ferrite nanoparticles decorated with gold nanoparticles exhibited smaller sizes with a slightly lower polydispersity. This was expected considering that core/shell NPs were obtained via growth of a gold shell around the gold-decorated NPs.

### 3.3. Photothermal Assays

In order to draw conclusions about the potential of these nanosystems for future applications in thermotherapy, several control assays were performed, namely, temperature assays (where the behavior of the nanosystems was assessed as a function of temperature variation) and irradiation assays (where the system components were irradiated with light, at constant temperature).

**Figure 12:**
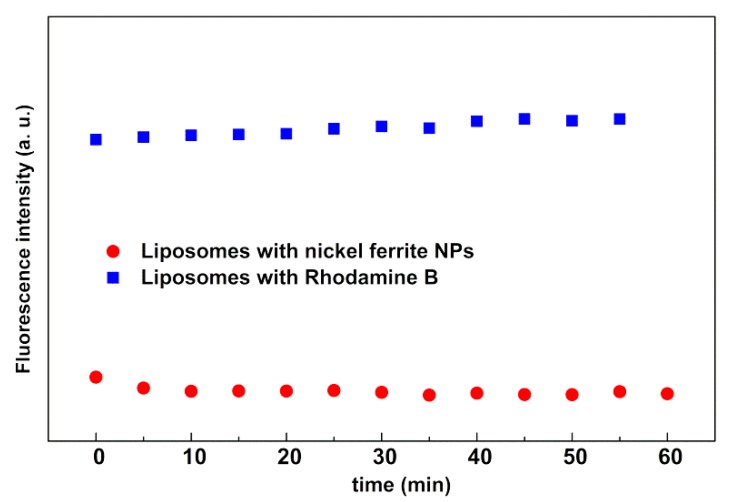
Fluorescence intensity as a function of time of irradiated liposomes containing nickel ferrite NPs (without gold) and liposomes containing only Rhodamine B-DOPE (without any NPs).

The irradiation assays were performed for the magnetoliposomes containing the magnetic nanoparticles without gold, liposomes containing only the fluorescent moiety, and the labeled lipid Rhodamine B-DOPE (without any NPs). The results, displayed in Figure 12, confirm that the irradiation had no effect on the nanosystems in the absence of gold, with these systems acting as negative controls. 

Considering, as an approximation, that the fluorescence quantum yield of Rhodamine B near the nanoparticles was much lower than the quenching ratio, $I_{F}^{0}/I_{F}$ (that is always higher than 1), the dependence of the fluorescence intensity ($I_{F}$) on temperature can be expressed as Equation (1) (detailed explanation in Supplementary Materials).

(1) $$\frac{I_{F}^{0}}{I_{F}}\propto A\;e^{-\;\frac{E_{a}}{RT}}\;$$

where A is the preexponential factor, *E_a_* is the activation energy, *R* is the gas constant, and *T* is the absolute temperature [49].

**Figure 13:**
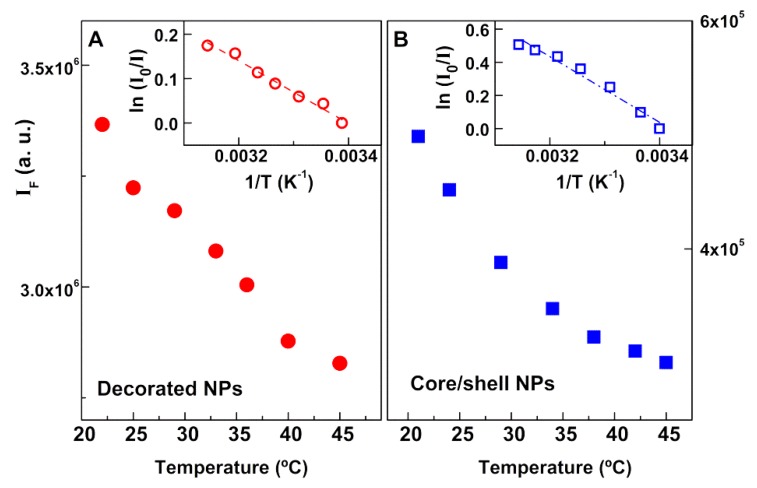
Fluorescence intensity of Rhodamine B as a function of temperature, for magnetoliposomes containing magnetic/plasmonic nanoparticles. Inset: Plots of$\;ln\left(I_{F}^{0}/I_{F}\right)$ versus *1/T.* (**A**) Decorated nanoparticles. (**B**) Core/shell nanoparticles.

This equation predicts an exponential decrease of the dye fluorescence intensity with increasing temperature. For magnetoliposomes containing magnetic/plasmonic NPs, the solution temperature was raised between room temperature and 45 °C, while the fluorescence intensity was monitored (Figure 13). The increase in temperature led to a decrease in fluorescence intensity of Rhodamine B, due to the enhancement of non-radiative deactivation pathways (decreasing the fluorescence quantum yield). A linear plot between$\;ln\left(I_{F}^{0}/I_{F}\right)$ and *1/T* was observed for both types of nanoparticles (gold-decorated nickel ferrite and core/shell nickel ferrite/gold) (insets of Figure 13).

**Figure 14:**
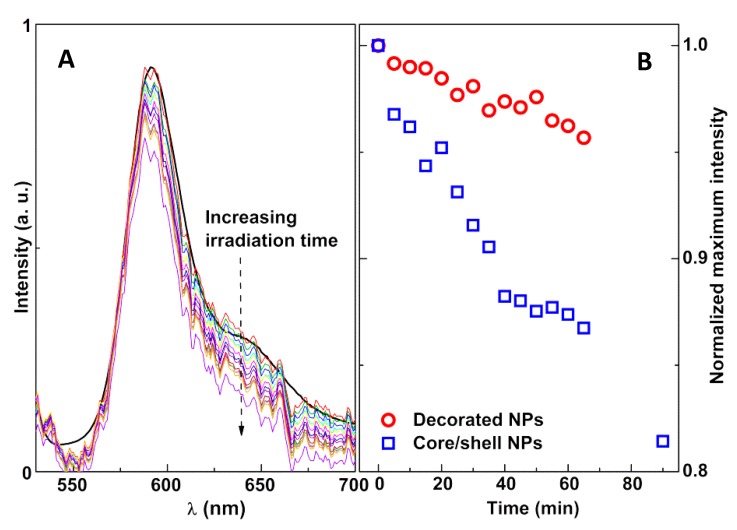
(**A**) Fluorescence spectra of Rhodamine B-DOPE in magnetoliposomes containing nickel ferrite/gold core/shell nanoparticles following several irradiation times (5-min steps, maximum: 90 min). The black line curve is the reference spectrum obtained in a commercial fluorimeter. (**B**) Variation of the normalized maximum intensity of Rhodamine B fluorescence with irradiation time, for magnetoliposomes containing both types of magnetic/plasmonic nanoparticles.

Upon irradiation, a similar behavior was observed, proving that a local rise in temperature originated from the plasmonic effect of gold, causing a decrease in Rhodamine fluorescence (Figure 14).

The photothermal effect was seen to be much more effective for the core/shell nanoparticles than for the decorated ones. This was expected, as gold nanoshells are frequently used in the photothermal ablation of cancer cells [19,50]. Using the linear plots in the insets of Figure 13, the local temperature rise was found to be 9.1 °C for the core/shell NPs and 5.5 °C for the gold-decorated nanoparticles. These values are an underestimation of the real photothermal capabilities of the studied systems as, due to experimental limitations, during the 1-min acquisition time of the Rhodamine B emission spectrum, the irradiation lamp was blocked. During that time, heat could diffuse away from the nanoparticles, leading to a local temperature decrease that was compensated for in the subsequent irradiation period. 

Compared with a previous work, where manganese ferrite/gold core/shell NPs were prepared using a different method [23], these nickel ferrite/gold NPs stand out, in the core/shell arrangement, as much more efficient in the increase of local temperature upon light absorption (λ > 600 nm) by the gold shell.

## 4. Conclusions

Magnetic/plasmonic nanoparticles of nickel ferrite/gold were prepared, including NiFe_2_O_4_ magnetic nanoparticles decorated with plasmonic Au nanoparticles and core/shell NiFe_2_O_4_/Au nanoparticles. These nanoparticles were covered with a surfactant/lipid (ODA/DOPG) bilayer, originating a liposome-like structure with sizes around 120–160 nm, suitable for therapeutic applications. The ability of the developed nanosystems to produce local heat was assessed, proving their promising utility for applications in thermotherapy.

## Figures and Tables

**Table 1:** X-ray diffraction Rietveld refinement calculated parameters R_f_ and χ^2^, phase sizes, and percentages. Au@NiFe_2_O_4_: gold-decorated nanoparticles; NiFe_2_O_4_/Au: core/shell nanoparticles.

Nanoparticles	Intensity Percentages		Phase Size (nm) Lattice Constant (Å)		Quality Parameters NiFe_2_O_4_|Au	
	NiFe_2_O_4_	Au	NiFe_2_O_4_	Au	R_f_	χ^2^
Au@NiFe_2_O_4_	89.1	10.9	11.5 8.331	22.7 4.071	5.16|3.81	1.43
NiFe_2_O_4_/Au	70.1	29.9	11.5 (*) 8.331 (*)	59.6 4.075	5.05|1.36	1.42

(*) fixed value.

**Table 2:** Coercive field (*H*_c_), saturation magnetization (*M*_s_), remnant magnetization (*M*_r_), and ratio *M*_r_/*M*_s_ for NiFe_2_O_4_/Au core/shell nanoparticles (NPs) and NiFe_2_O_4_ NPs decorated with gold, at room temperature.

Nanoparticles	*H*_c_ (Oe)	*M*_r_ (emu/g)	*M*_s_ (emu/g)	*M*_r_/*M*_s_
NiFe_2_O_4_ NPs decorated with Au NPs	29.00	0.61	20.55	0.030
NiFe_2_O_4_/Au core/shell NPs	17.19	0.06	2.65	0.023

**Table 3:** Size and polydispersity of solid magnetoliposomes (SMLs) determined by dynamic light scattering (DLS) (SD: standard deviation; PDI: polydispersity index).

Nanoparticles	Size ± SD (nm)	PDI ± SD
Nickel ferrite decorated with gold NPs	118.5 ± 8	0.171 ± 0.03
Nickel ferrite/gold core/shell NPs	158 ± 12	0.184 ± 0.05

## Supplementary Materials

The following are available online at https://www.mdpi.com/1996-1944/13/4/815/s1: Figure S1: UV–Vis maximum absorbance of nanoparticles dispersions in PBS buffer (pH = 7.0) as function of time (5-min intervals). Fluorescence intensity dependence on temperature in irradiation assays.
