# Supplementary material for: Development of Novel Magnetoliposomes Containing Nickel Ferrite Nanoparticles Covered with Gold for Applications in Thermotherapy
Source: Materials (Basel). 2020 Feb 11;13(4):815. doi: 10.3390/ma13040815 (PMC7078757; doi:10.3390/ma13040815)
Supplement: Supplementary file 1 [file materials-13-00815-s001.pdf]

materials

Supplementary Material

Development of Novel Magnetoliposomes Containing Nickel Ferrite Nanoparticles Covered with Gold for Applications in Thermotherapy

Irina S. R. Rio [1], Ana Rita O. Rodrigues [1], Carolina P. Rodrigues [1], Bernardo G. Almeida [1], A. Pires[2], A. M. Pereira [2], J. P. Araújo [2], Elisabete M. S. Castanheira [1] and Paulo J. G. Coutinho [1,*]

Evidence of stability of NPs dispersions

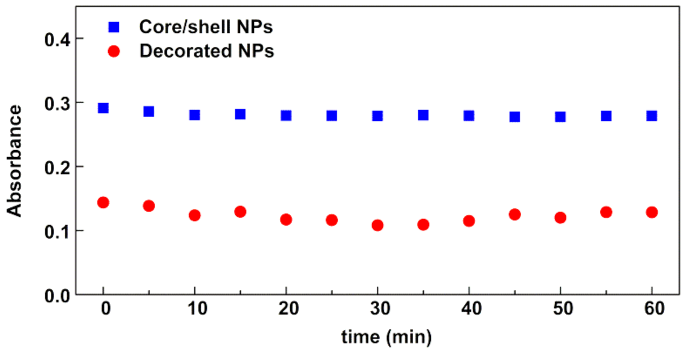

Figure S1. UV-Visible maximum absorbance of nanoparticles dispersions in PBS buffer (pH = 7.0) as function of time (5 minutes intervals).

Fluorescence intensity dependence on temperature in irradiation assays

Assuming an Arrhenius behaviour for the rate constant, k_{nr}, of non-radiative processes, it is possible to determine the temperature in each irradiation assay,

$$k_{nr} = k_0 e^{-\frac{E_a}{RT}} \tag{S1}$$

where k_0 is the preexponential factor; E_a is the activation energy, R the gas constant and T the absolute temperature.

The fluorescence quantum yield, Φ_F, is given by

$$\Phi_F = \frac{k_F}{k_F + k_{nr}} \Rightarrow \frac{1}{\Phi_F} - 1 = \frac{k_{nr}}{k_F} \tag{S2}$$

where k_F is the fluorescence rate constant.

Taking as reference the fluorescence quantum yield at room temperature, Φ_F^0, it is obtained

$$\frac{\Phi_F^0}{\Phi_F} - \Phi_F^0 = \Phi_F^0 \frac{k_{nr}}{k_F} \tag{S3}$$

or, similarly,

$$\frac{I_F^0}{I_F} - \Phi_F^0 = \Phi_F^0 \frac{k_{nr}}{k_F} \tag{S4}$$

where I_F represents the fluorescence intensity at a given wavelength.

Therefore, it can be obtained

$$\frac{I_F^0}{I_F} - \Phi_F^0 = \Phi_F^0 \frac{k_0}{k_F} e^{-\frac{E_a}{RT}} \tag{S5}$$

Considering, as an approximation, that the fluorescence quantum yield of Rhodamine B near the nanoparticles is much lower than the quenching ratio, $\frac{I_F^0}{I_F}$, that is always above unity, it is obtained:

$$\frac{I_{\mathrm{F}}^0}{I_{\mathrm{F}}} \approx \Phi_{\mathrm{F}}^0 \frac{k_0}{k_{\mathrm{F}}} e^{-\frac{E_a}{RT}} \tag{S6}$$

Or

$$\frac{I_{\mathrm{F}}^0}{I_{\mathrm{F}}} \propto A\, e^{-\frac{E_a}{RT}} \tag{S7}$$

© 2020 by the authors. Submitted for possible open access publication under the terms and conditions of the Creative Commons Attribution (CC BY) license (http://creativecommons.org/licenses/by/4.0/).
